# Supplementary figures and images for: Global change impacts on bird biodiversity in South Asia: potential effects of future land-use and climate change on avian species richness in Pakistan
Source: PeerJ. 2023 Oct 6;11:e16212. doi: 10.7717/peerj.16212 (PMC10561643; doi:10.7717/peerj.16212)

# Elevation

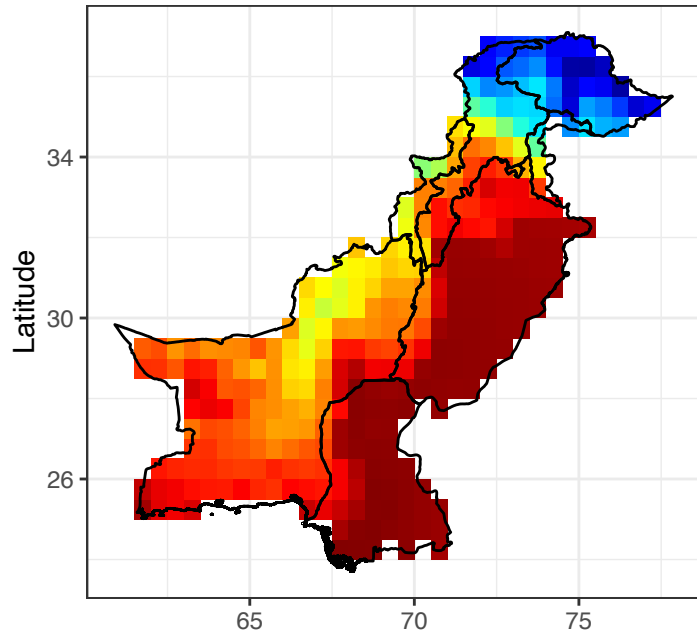

## Elevation [m]

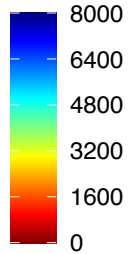

# Population

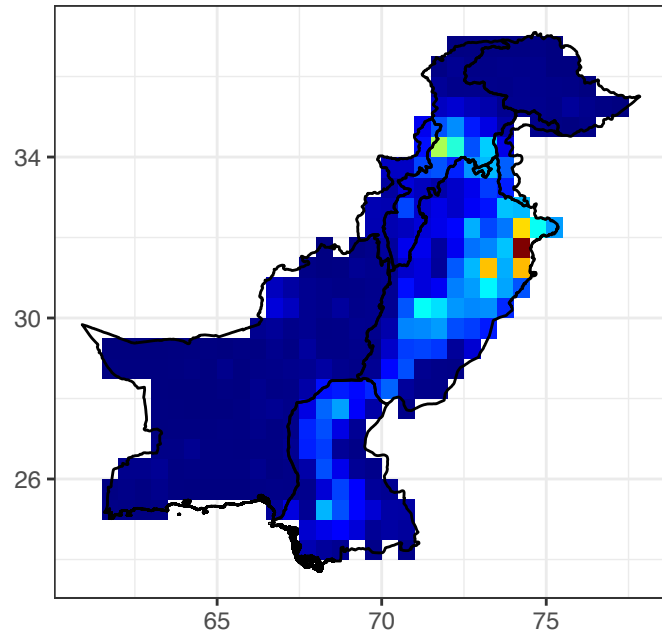

## Population

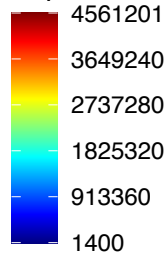

Longitude

Supplement: Supplemental Information 1 — The internal borders indicate the borders of the provinces. [file peerj-11-16212-s001.pdf]

Breeding birds temperature

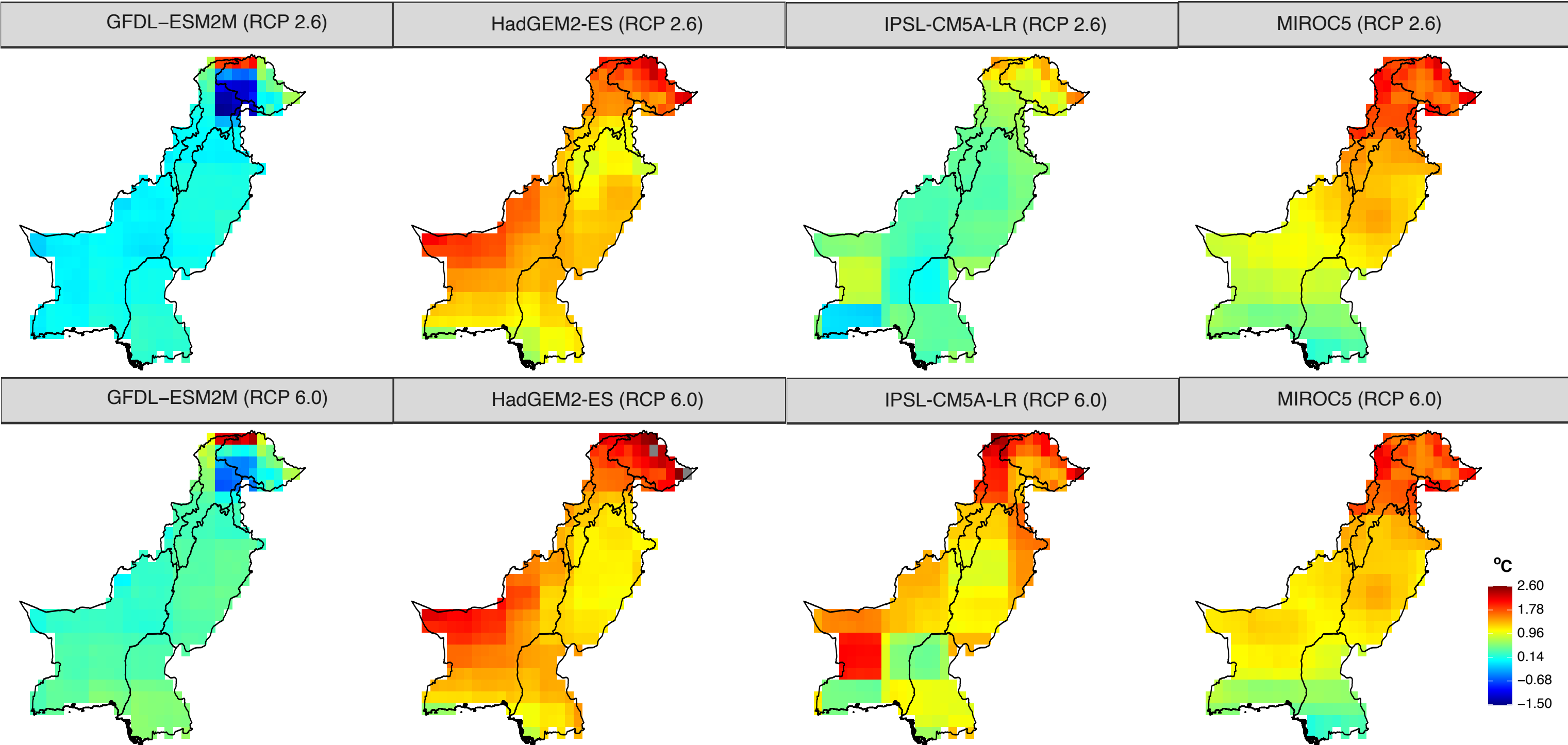

Wintering birds temperature

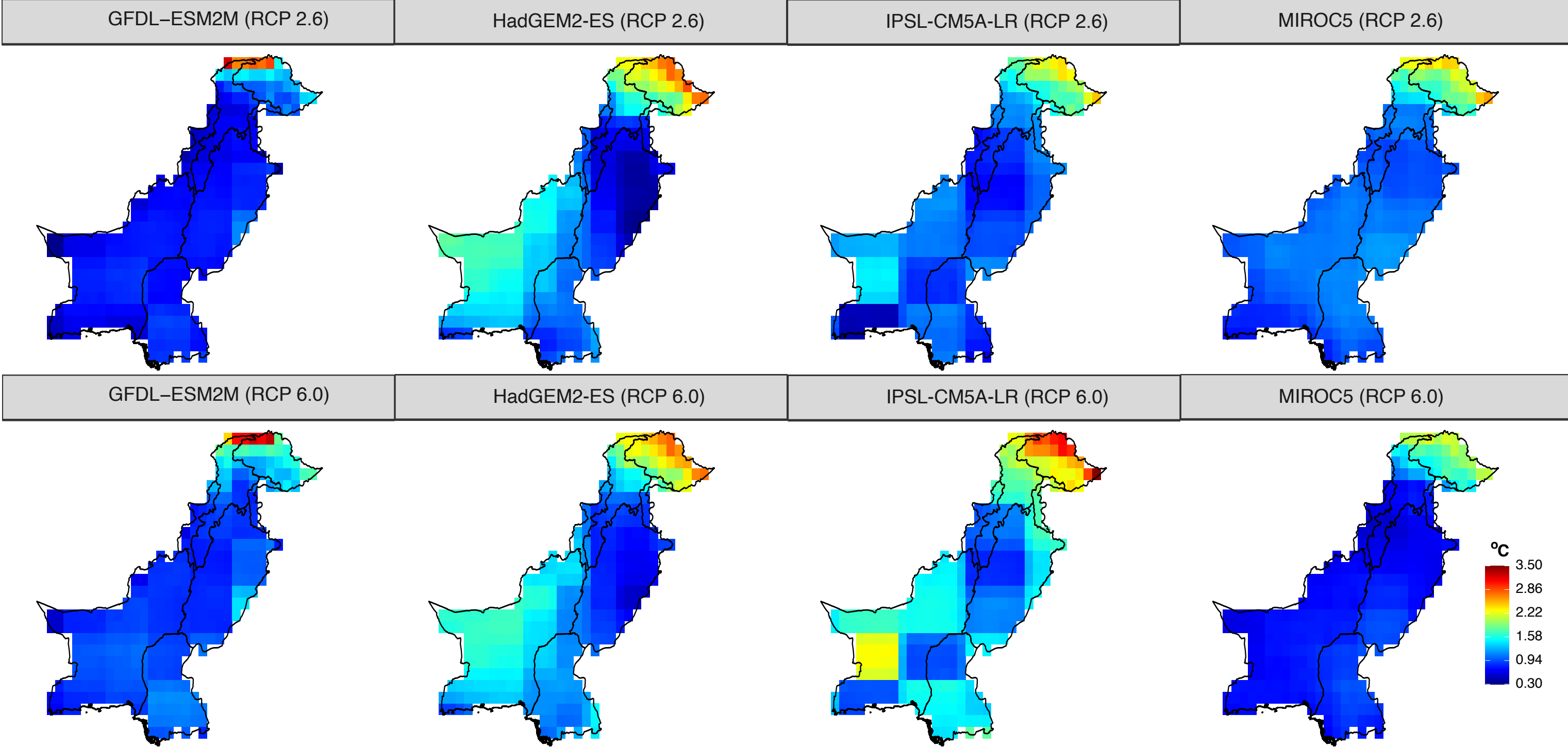

Supplement: Supplemental Information 2 — Exposure is calculated as the difference between mean of current conditions (1980–2010) and mean of future conditions (2035–2065) in each grid cell of 0.5 × 0.5° resolution. Units are °C. [file peerj-11-16212-s002.pdf]

Summer precipitation change

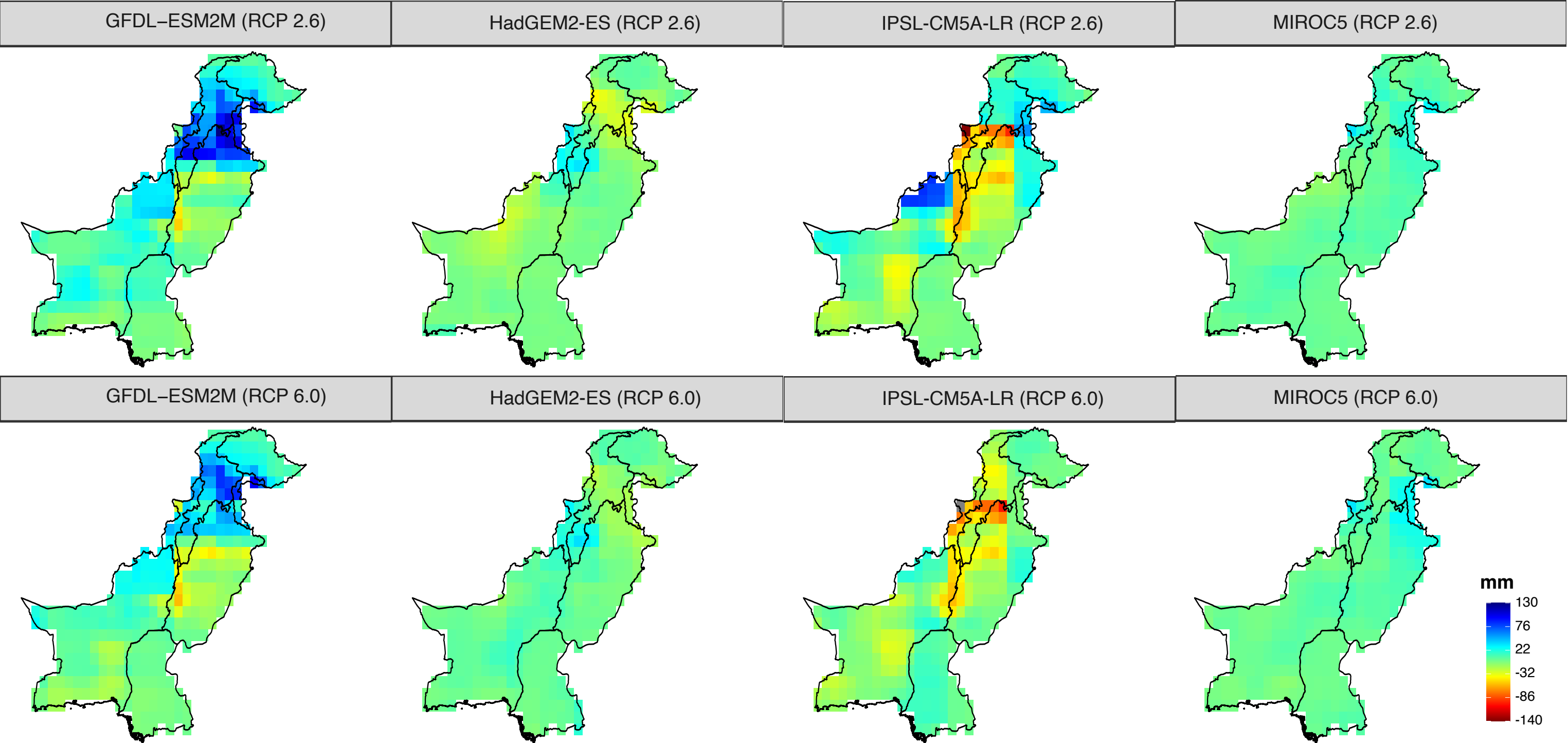

Winter precipitation change

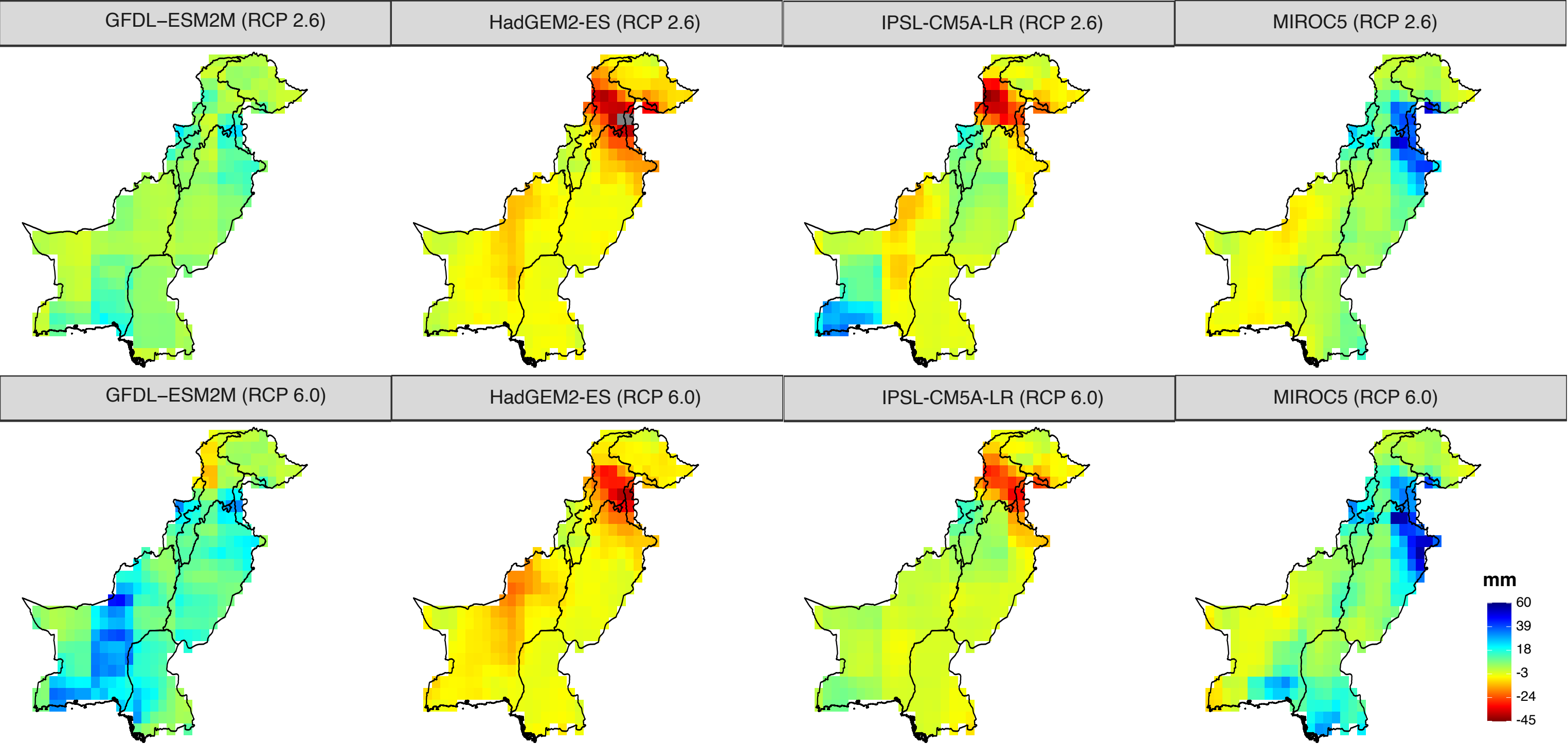

Supplement: Supplemental Information 3 — Exposure is calculated as the difference between mean of current conditions (1980–2010) and mean of future conditions (2035–2065) in each grid cell of 0.5 × 0.5° resolutions. Units are in mm. [file peerj-11-16212-s003.pdf]

Combined impact, breeding birds

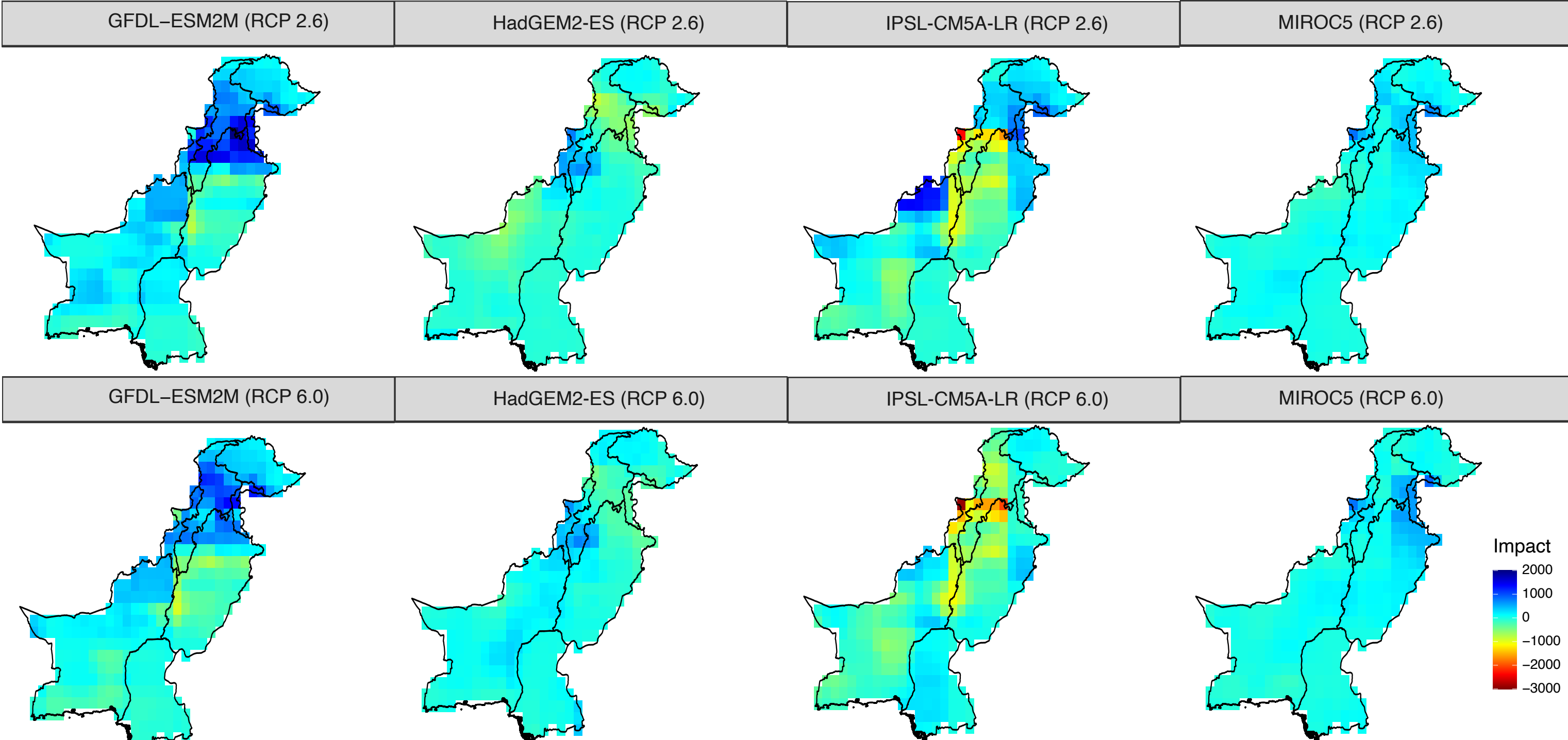

Combined impact, wintering birds

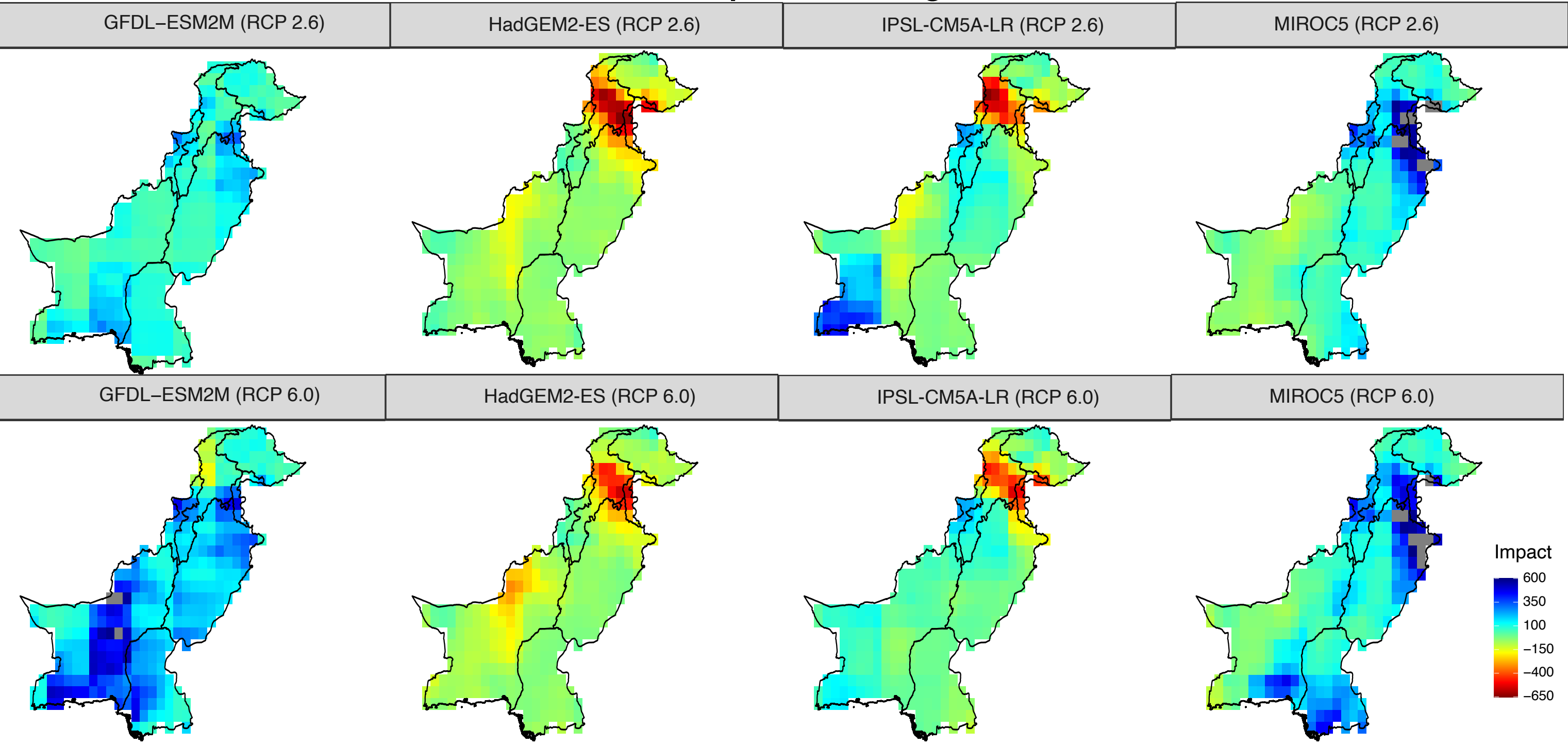

Supplement: Supplemental Information 5 — Impact of mean seasonal temperature, total seasonal precipitation and cropland is calculated as the product of slope of each variable in regression model (i.e., species sensitivity) and difference between mean of current conditions (1980–2010) and mean of future conditions (2035–2065) (i.e., species exposure) of each variable in each grid cell of 0.5 × 0.5° resolution. For temperature and precipitation, we calculated the combined impact for future scenario RCP2.6 and RCP6 for four different general circulation models (GCMs). Units are number of species. [file peerj-11-16212-s005.pdf]
