# Supplementary material for: Global change impacts on bird biodiversity in South Asia: potential effects of future land-use and climate change on avian species richness in Pakistan
Source: PeerJ. 2023 Oct 6;11:e16212. doi: 10.7717/peerj.16212 (PMC10561643; doi:10.7717/peerj.16212)
Supplement: Supplemental Information 4 — Exposure is calculated as the difference between mean of current conditions (1980–2010) and mean of future conditions (2035–2065) in each grid cell of 0.5 × 0.5° resolution. Units are in percentage. [file peerj-11-16212-s004.pdf]

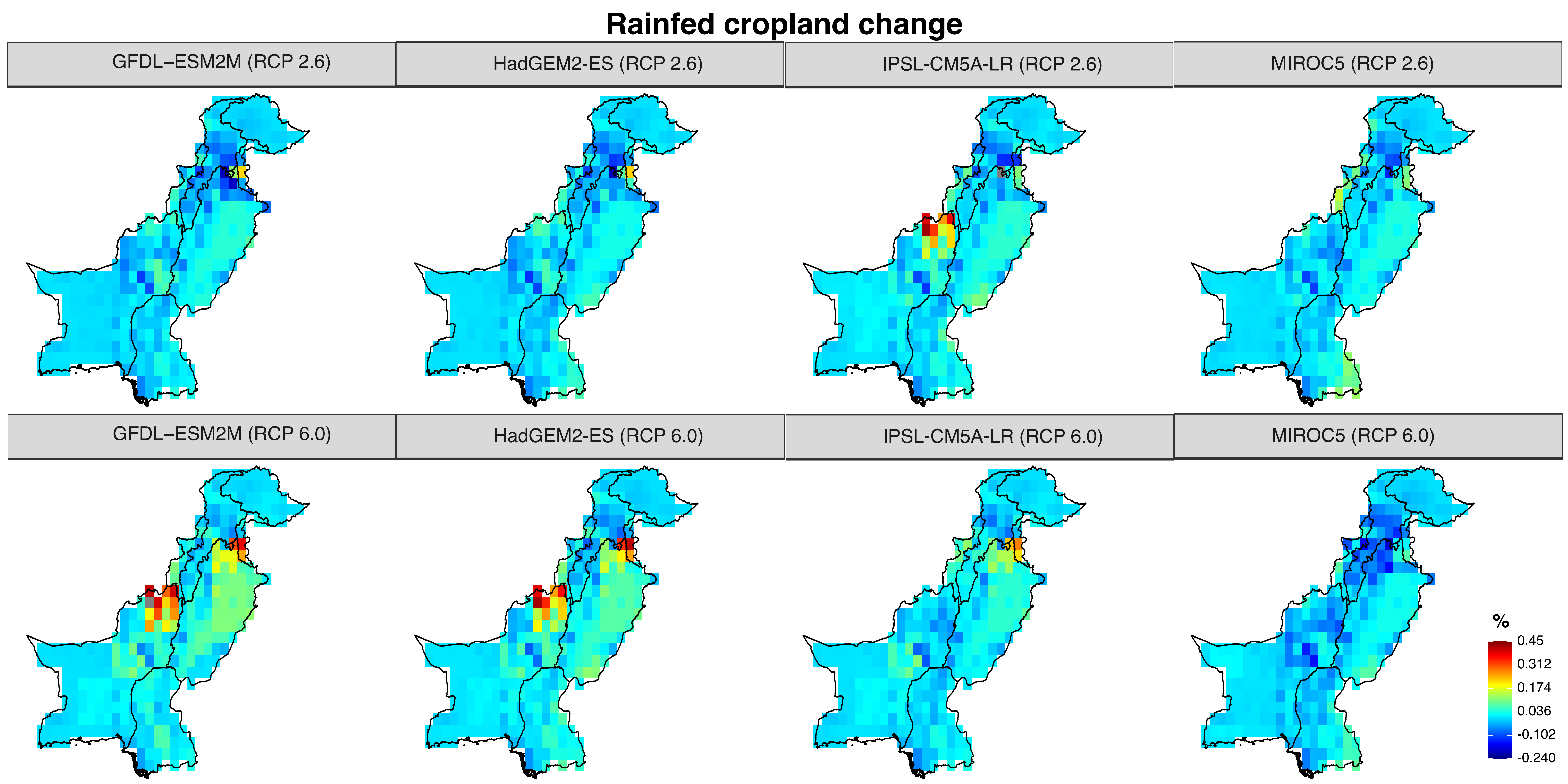
Rainfed cropland change
GFDL–ESM2M (RCP 2.6)
HadGEM2-ES (RCP 2.6)
IPSL-CM5A-LR (RCP 2.6)
MIROC5 (RCP 2.6)
GFDL–ESM2M (RCP 6.0)
HadGEM2-ES (RCP 6.0)
IPSL-CM5A-LR (RCP 6.0)
MIROC5 (RCP 6.0)
%
0.45
0.312
0.174
0.036
-0.102
-0.240
