# Supplementary material for: Global change impacts on bird biodiversity in South Asia: potential effects of future land-use and climate change on avian species richness in Pakistan
Source: PeerJ. 2023 Oct 6;11:e16212. doi: 10.7717/peerj.16212 (PMC10561643; doi:10.7717/peerj.16212)
Supplement: Supplemental Information 8 — Exposure of temperature (summer and winter) for different scenarios and global climate models (GCMs). Exposure is calculated as the difference between mean of current conditions (1980–2010) and mean of future conditions (2035–2065) in each grid cell of 50 by 50 equal area grid cell. [file peerj-11-16212-s008.docx]

**Global change impacts on bird biodiversity in South Asia: potential effects of future land-use and climate change on avian species richness in Pakistan**

Imran Khaliq^1,2^, Matthias F. Biber^3^, Diana Bowler^4^ and Christian Hof^3^.

Supplementary Information:

**Table S1** Association of species richness of breeding and wintering birds of Pakistan with climate, land-use change, water discharge in rivers and human population. Species richness of breeding birds and wintering birds was modeled as function of mean maximum seasonal temperature, total seasonal precipitation, maximum water discharge, pastures, rainfed cropland, irrigated cropland and human population in a generalized least squares model while adding the spatial covariance structure in the variance-covariance matrix. We also added interaction terms between temperature, precipitation and rainfed cropland. All variables are averaged over 0.5 × 0.5° resolution. Variables were kept in their original units for interpretation.

| **Breeding birds** | | | | |
| --- | --- | --- | --- | --- |
|  | β | Std. error | t Value | P-value |
| Mean seasonal maximum temperature (°C) | **9.306** | **±2.89** | **3.20** | **0.001** |
| Total seasonal precipitation (mm) | **14.91** | **±3.05** | **4.88** | **<0.001** |
| Maximum water discharge from rivers (m^3^s-1) | -0.08 | ±0.43 | -0.18 | 0.85 |
| Pastures (%) | 0.74 | ±0.77 | 0.96 | 0.33 |
| Rainfed Cropland (%) | **4.67** | **±1.08** | **4.31** | **<0.001** |
| Irrigated Cropland (%) | 0.66 | ±1.08 | 0.60 | 0.54 |
| Human population | -0.06 | ±0.82 | -0.08 | 0.93 |
| **Temperature:Precipitation** | **3.83** | **1.45** | **2.62** | **0.009** |
| **Temperature:Rainfed cropland** | **-4.94** | **1.54** | **-3.19** | **0.001** |
| Precipitation:Rainfed cropland | -1.35 | 1.00 | -1.35 | 0.17 |
| Temperature:Precipitation:Rainfed cropland | 0.68 | 0.80 | 0.84 | 0.39 |
| **Winter birds** | | | | |
|  | β | Std. error | t Value | P-value |
| Mean seasonal maximum temperature (°C) | **20.29** | **±3.23** | **6.28** | **<0.001** |
| Total seasonal precipitation (mm) | **10.29** | **±2.68** | **3.84** | **<0.001** |
| Maximum water discharge from rivers (m^3^s-1) | 0.29 | ±0.45 | 0.65 | 0.51 |
| Pastures (%) | 0.18 | ±0.18 | 0.22 | 0.82 |
| Rainfed Cropland (%) | **4.08** | **±1.15** | **3.56** | **<0.001** |
| Irrigated Cropland (%) | 0.64 | ±1.17 | 0.54 | 0.58 |
| Human population | 0.006 | ±0.007 | 0.007 | 0.99 |
| **Temperature:Precipitation** | **2.77** | **1.37** | **2.02** | **0.04** |
| Temperature:Rainfed cropland | -2.88 | 1.65 | -1.74 | 0.08 |
| Precipitation:Rainfed cropland | -0.66 | 0.92 | -0.72 | 0.46 |
| Temperature:Precipitation:Rainfed cropland | 1.28 | 0.88 | 1.46 | 0.14 |


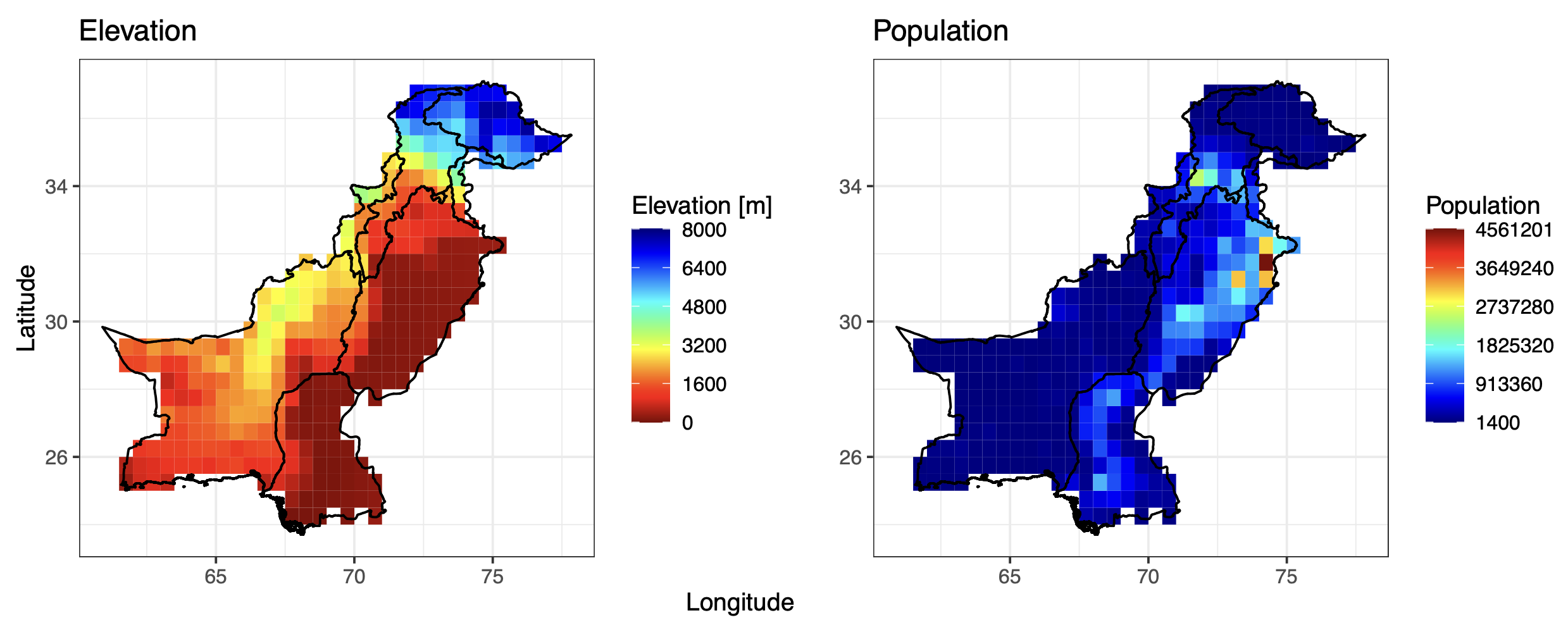


**Fig. S1** Distribution of elevation (m) and human population across Pakistan. The internal borders indicate the borders of the provinces.


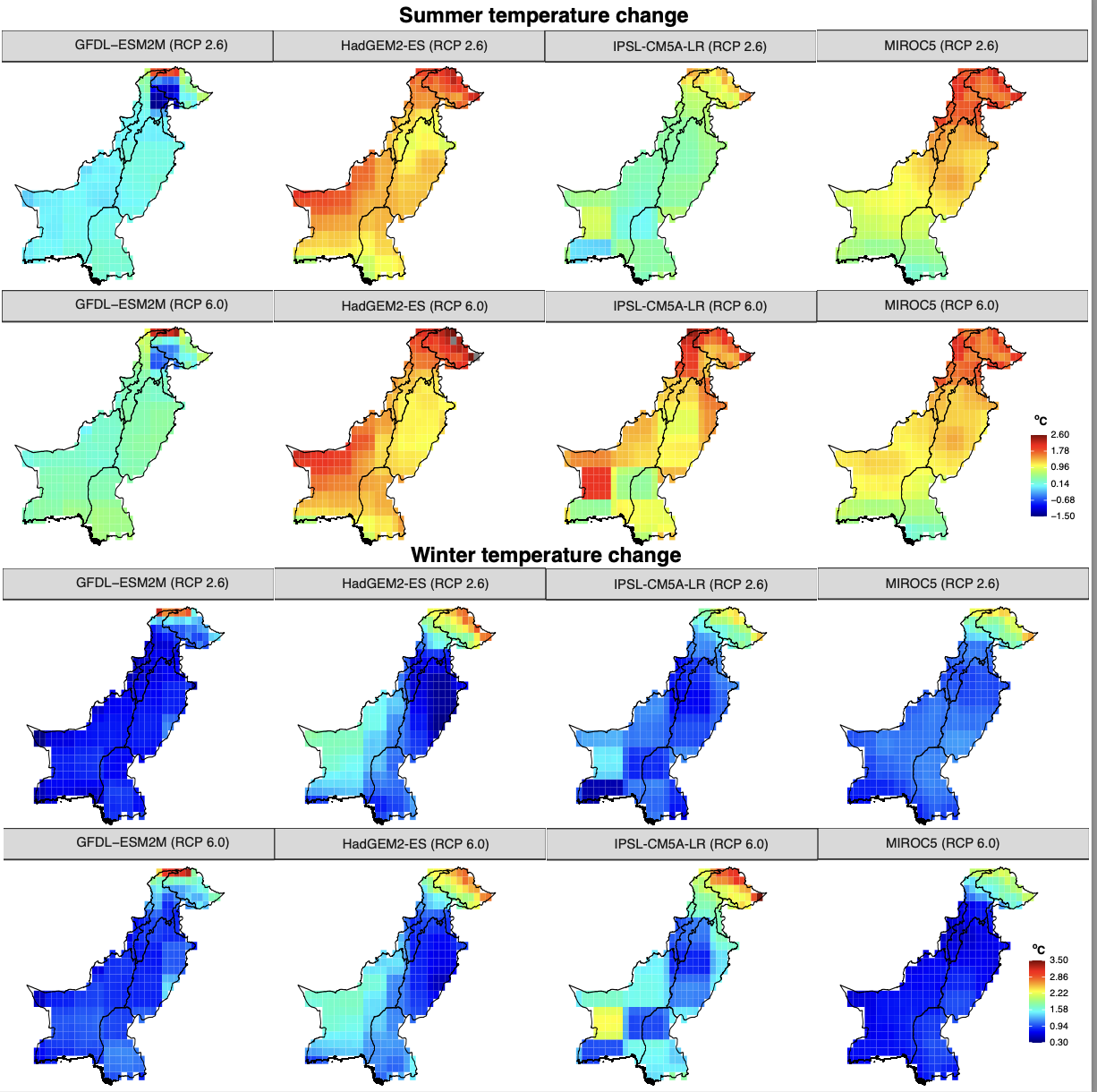


**Fig. S2** Exposure of mean seasonal temperature change (summer and winter) for different scenarios and global climate models (GCMs). Exposure is calculated as the difference between mean of current conditions (1980-2010) and mean of future conditions (2035-2065) in each grid cell of 0.5 × 0.5° resolution. Units are ^o^C.


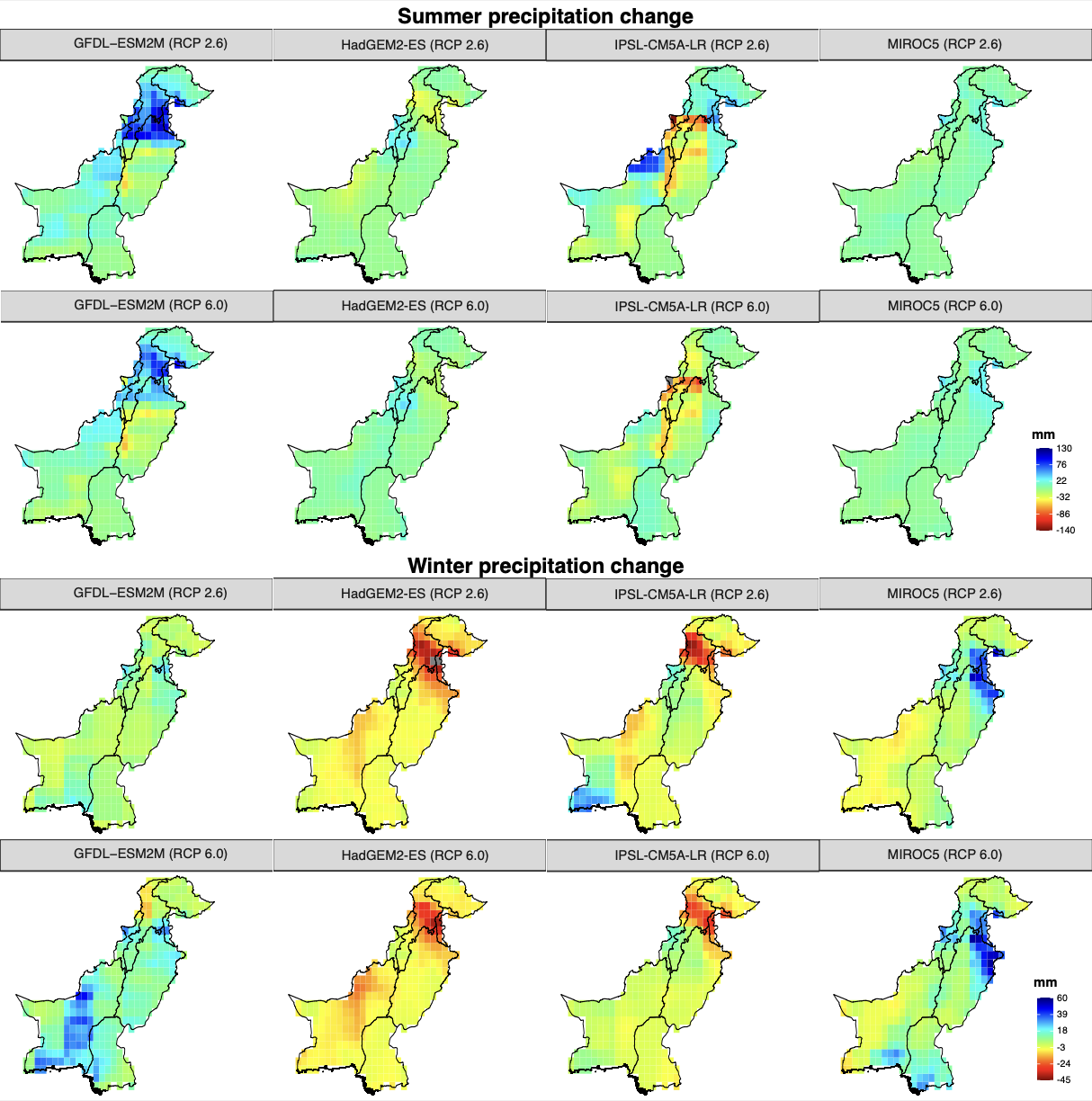


**Fig. S3** Exposure of total seasonal precipitation change (summer and winter) for different scenarios and global climate models (GCMs). Exposure is calculated as the difference between mean of current conditions (1980-2010) and mean of future conditions (2035-2065) in each grid cell of 0.5 × 0.5° resolutions. Units are in mm.


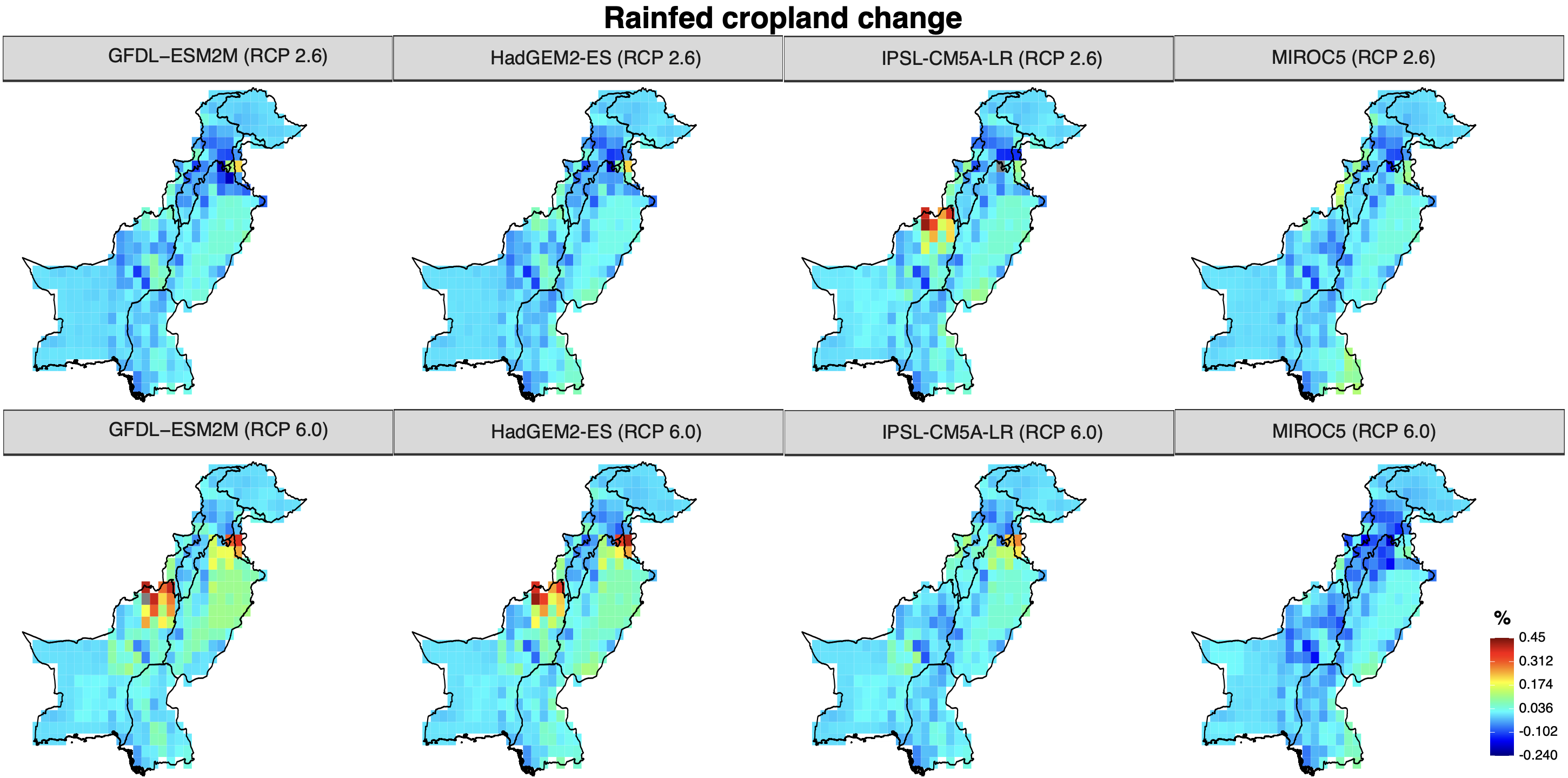


**Fig. S4** Exposure of rainfed cropland (summer and winter) for different scenarios and global climate models (GCMs). Exposure is calculated as the difference between mean of current conditions (1980-2010) and mean of future conditions (2035-2065) in each grid cell of 0.5 × 0.5° resolution. Units are in percentage.


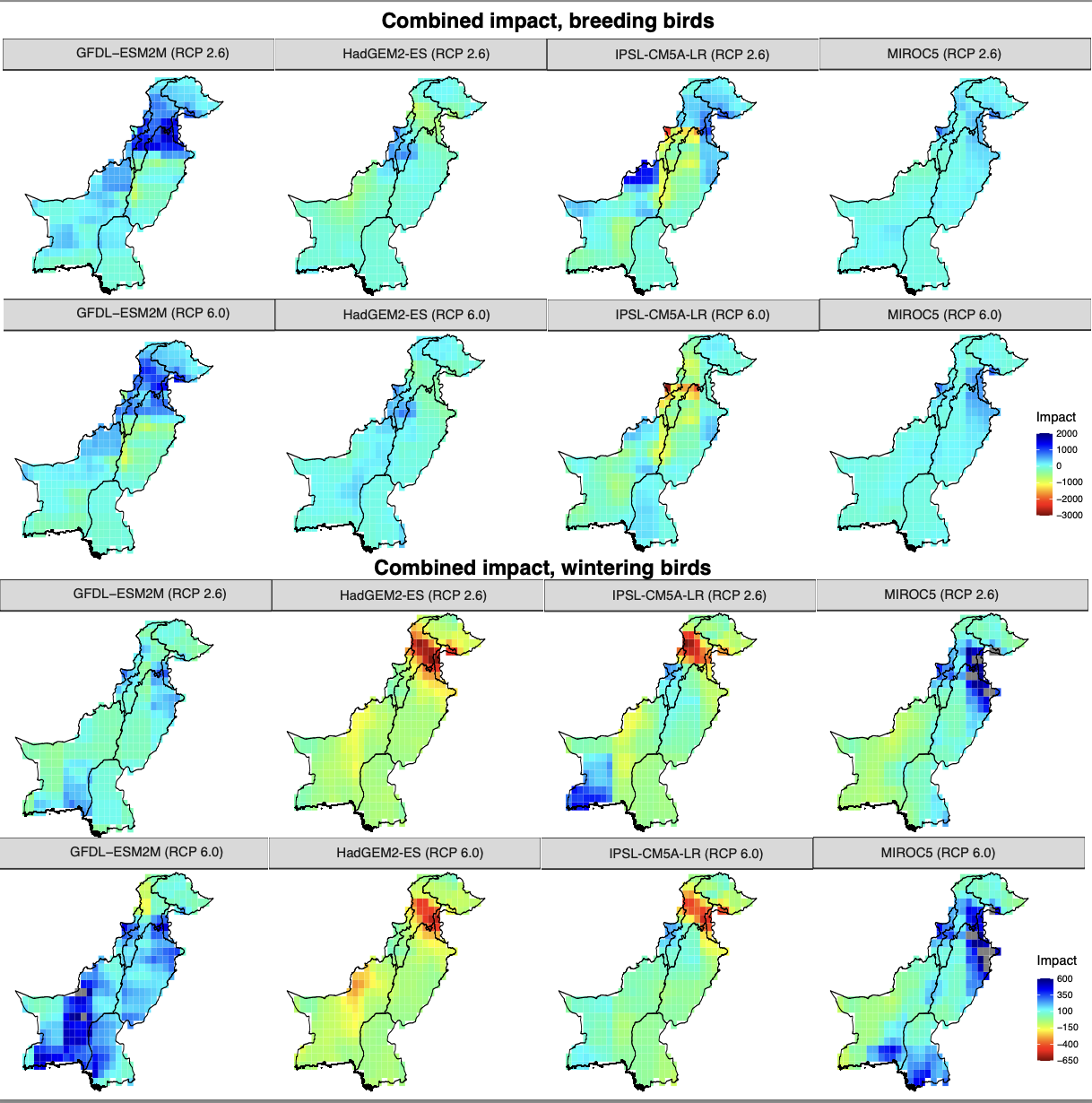


**Fig. S5** Figure showing the combined impact of significant drivers of species richness. Impact of mean seasonal temperature, total seasonal precipitation and cropland is calculated as the product of slope of each variable in regression model (i.e., species sensitivity) and difference between mean of current conditions (1980-2010) and mean of future conditions (2035-2065) (i.e., species exposure) of each variable in each grid cell of 0.5 × 0.5° resolution. For temperature and precipitation, we calculated the combined impact for future scenario RCP2.6 and RCP6 for four different general circulations models (GCMs). Units are number of species.
